# Sarcoidosis in tuberculosis-endemic regions: India

**DOI:** 10.1186/1869-5760-3-53

**Published:** 2013-06-27

**Authors:** Kalpana Babu

**Affiliations:** 1 Vittala International Institute of Ophthalmology, Hosakerehalli, Bangalore, Karnataka, 560085, India; 2 Prabha Eye clinic and Research Center, 40th Cross Rd, Jayanagar 8th Block, Bangalore, 560070, India

**Keywords:** Sarcoidosis, Tuberculosis, Ocular involvement, Computed tomography, Mantoux test

## Abstract

Sarcoidosis is a multisystem inflammatory disease of unknown etiology affecting multiple organs. Earlier reports suggested that sarcoidosis was a disease of the developed world. However, recent reports suggest that the disease is found in the developing countries as well. Clinical, radiological, and histopathological similarities with tuberculosis pose a great challenge in countries endemic for tuberculosis. Mantoux test, high resolution computed tomography, and transbronchial lymph node and lung biopsies are diagnostic modalities, which play an important role in the diagnosis of sarcoid. In this review, we look at the epidemiology of sarcoid in tuberculosis-endemic regions, the sarcoidosis-tuberculosis link, clinical profile, diagnostic modalities, dilemma in the diagnosis, and the treatment of this disease.

## Review

### Introduction

Sarcoidosis is a chronic, multisystem inflammatory disease of unknown etiology characterized by the presence of non-caseating granulomas in different organs. Although the lungs are the most common sites of inflammation, sarcoidosis can involve other organs such as the eyes (intraocular and adnexal), skin, lymph nodes, salivary glands, heart, spleen, liver, and the nervous system [1]. A combination of clinical, radiological, and histological criteria are used to diagnose sarcoidosis. Remarkable clinical similarities with tuberculosis (TB) make the differential diagnosis of the two conditions difficult especially in countries with high burden of tuberculosis. Negative Mantoux test and exclusion of mycobacterial infection or caseation in the biopsy specimens help in diagnosing sarcoidosis. Among the high TB endemic countries, there is increasing literature available on sarcoidosis from India. In this review, we look at the tuberculosis link, epidemiology of sarcoidosis in areas with high TB burden especially India, the emerging literature on sarcoidosis in high TB endemic areas like India, the clinical profile of sarcoidosis including the systemic and ocular features, the diagnostic modalities, and the dilemma faced in the diagnosis and treatment.

### Epidemiology of sarcoidosis in areas with high TB burden

Sarcoidosis was considered to be a disease seen more commonly in the west and rarely in the developing world in earlier reports. In a study from eight countries of Asia and Africa in 1976, fewer than 30 patients were reported from India, Malaysia, Thailand, Taiwan, and United Arab Emirates, while none from Singapore and Korea [2]. In the recent years, there has been increasing reports from countries like India [3-13], Singapore [14], Malaysia [15], Thailand [16], and Taiwan [17]. In all probabilities, the disease was probably overshadowed by the presence of tuberculosis. Reports have also mentioned that the increase in incidence of sarcoidosis was probably related to the decrease in infectious diseases, especially tuberculosis and the implementation of tuberculosis control programs [4]. However, the cases of tuberculosis are still prevalent in the developing world, but we diagnose sarcoidosis with greater frequency in recent times. Unlike in the past, this is probably due to the increased awareness of the disease and improved efforts to make a diagnosis. The availability of computed tomography and fiberoptic bronchoscopy for transbronchial lung and lymph node biopsies has made a big difference in the diagnosis of the disease [12].

The true burden of sarcoidosis in India is not clearly known, as reliable epidemiological data are not available. It has been estimated that sarcoidosis constituted 10 to 12 cases per 1,000 new registrations in a respiratory unit at Kolkata and 61.2/100,000 new cases at a center in New Delhi in a report published in 2002 [13]. The cases of sarcoidosis have been reported across the country with no data on regional or ethnic differences and are now considered routinely in the differential diagnosis of respiratory and non-respiratory diseases including ocular problems. Reports of ocular sarcoid have been reported from across the country in the recent years [8-10,18-22].

### The tuberculosis link

Mycobacterium tuberculosis as a cause of sarcoidosis has been extensively studied in the literature [23-25]. Reports of previous history of contact with tuberculosis in patients with sarcoidosis [26], similarities in time trends in the prevalence of the two diseases, and ethnic populations with higher incidence of TB having high incidence of sarcoidosis are described in the literature [27,28]. Studies from India reporting patients with TB preceding the development of sarcoidosis or concurrent presence of both diseases have been described [3,4]. Molecular techniques such as nucleic acid amplification tests have demonstrated mycobacterial DNA and RNA in 30% of sarcoid biopsy tissues [5,25]. This was 48% in a prospective study from India [5]. Mycobacterial proteins like *MTb* catalase peroxidase (mKatG), superoxide dismutase, mycolyl transferase, and heat shock proteins have been detected [29]; immune responses to them have also been detected in sarcoid tissues, blood, and bronchoalveolar lavage fluid. When interferon-gamma (IFN-γ) ELISPOT assays and flow cytometry were used to assess the lung and blood T-cell responses to mKatG in sarcoidosis patients, a higher frequency of mKatG-reactive, IFN-γ expressing T cells was demonstrated in patients with active sarcoidosis than in controls [30-33]. This antigen also elicited granulomatous response in animal models, renewing interest in mycobacteria as a causative agent in sarcoidosis. Studies from India have shown positivity to QuantiFERON TB gold test in a small subset of sarcoid patients [6].

### Systemic sarcoidosis: clinical features

The course of sarcoidosis ranges from asymptomatic to severe disease. Male preponderance with a mean age of 40 years is reported in most Indian reports [3,4,7,10]. Reports in ophthalmology literature from south India have shown a female preponderance [9].

The disease affects predominantly the lungs, thoracic lymph nodes, skin, and eyes. Clinically, patients may be asymptomatic or complain of cough or breathlessness. Constitutional symptoms such as fever, malaise, fatigue, and weight loss may be present. The most frequent intrathoracic involvement is the presence of hilar or mediastinal lymph node enlargements with or without radiologic evidence of interstitial involvement in one-third of patients. The miliary pattern can be seen in sarcoidosis and may be misdiagnosed and treated for tuberculosis [3]. High resolution computed tomography (CT) is more diagnostic than chest X-ray [3,4,22].

Skin involvement may include sarcoid granulomas and erythema nodosum. Bilateral parotid gland enlargement, hepatic involvement with granulomas, renal insufficiency, myocardial involvement with heart failure, hypertension and conduction abnormalities, arthropathy, neurological involvement in the form of recurrent laryngeal nerve palsy, Bells' palsy, other cranial neuropathies and spinal chord involvement, generalized lymphadenopathy, and minor hematologic abnormalities have been reported from India [3,4]. Sporadic reports of childhood sarcoidosis are seen in India. Those cases manifesting with generalized lymphadenopathy are mistaken for TB and are usually treated initially with antitubercular therapy [3].

### Ocular sarcoidosis: clinical features

Patients may present directly to an ophthalmology clinic with blurred vision, floaters, redness or discomfort, or may be referred by the pulmonologists or rheumatologists for an ocular evaluation. Ocular disease may be the initial manifestation in sarcoidosis. Ocular involvement occurs in 25% to 60% of patients with systemic sarcoidosis presenting to a respiratory center [10]. This was around 95% in those who presented to the tertiary eye care center probably due to the referral bias [9]. Ocular manifestations include uveitis, dry eye, conjunctival granulomas, and involvement of the orbit and optic nerve [9,10,18-21,34].

In contrast to reports from western literature [35], panuveitis is the most common manifestation seen in most of the reports from India. As most of the reports are from tertiary referral centers, it could be possible that initial presentations of anterior uveitis could be treated by general ophthalmologists prior to referral.

**Figure 1:**
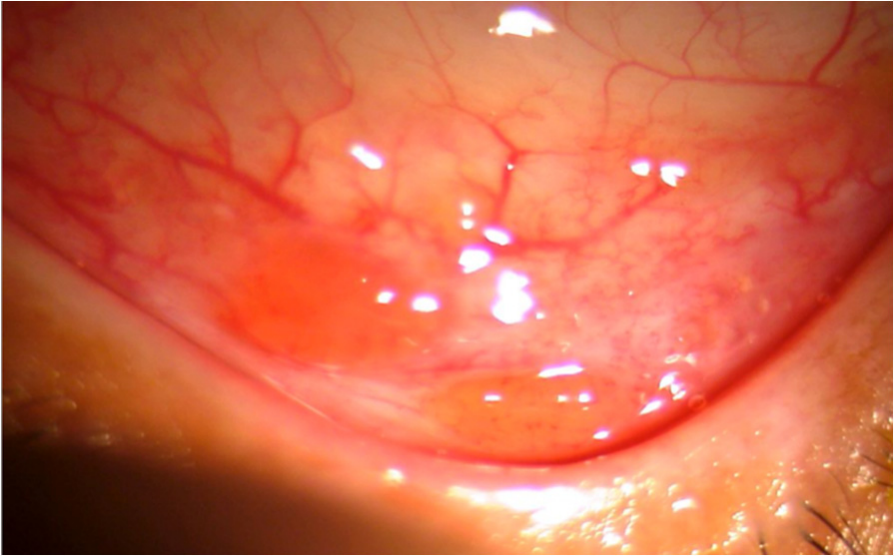
Slit lamp photograph showing the conjunctival granulomas in a patient with thoracic sarcoid.

**Figure 2:**
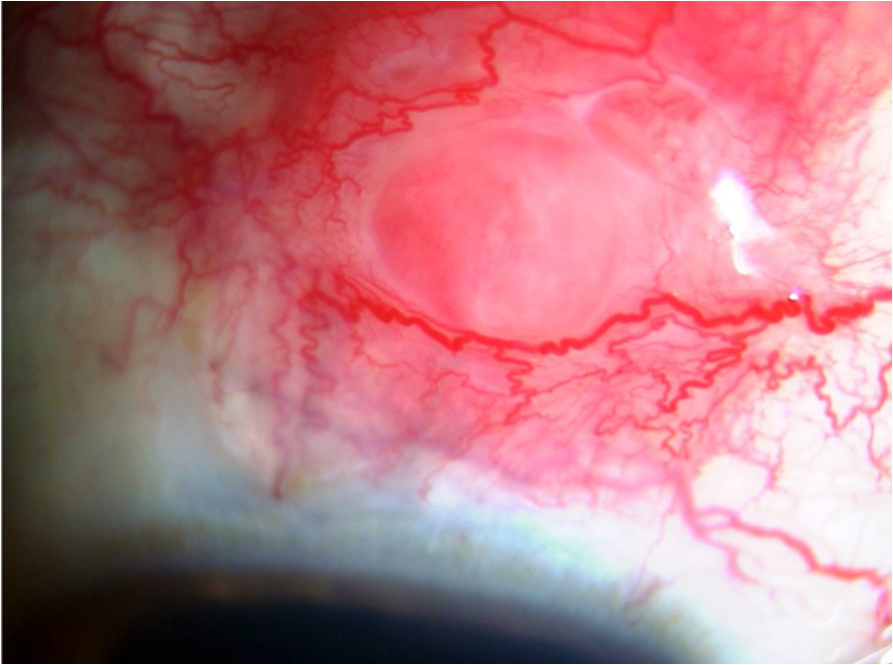
Slit lamp photograph showing the scleral nodule in a patient with sarcoidosis.

**Figure 3:**
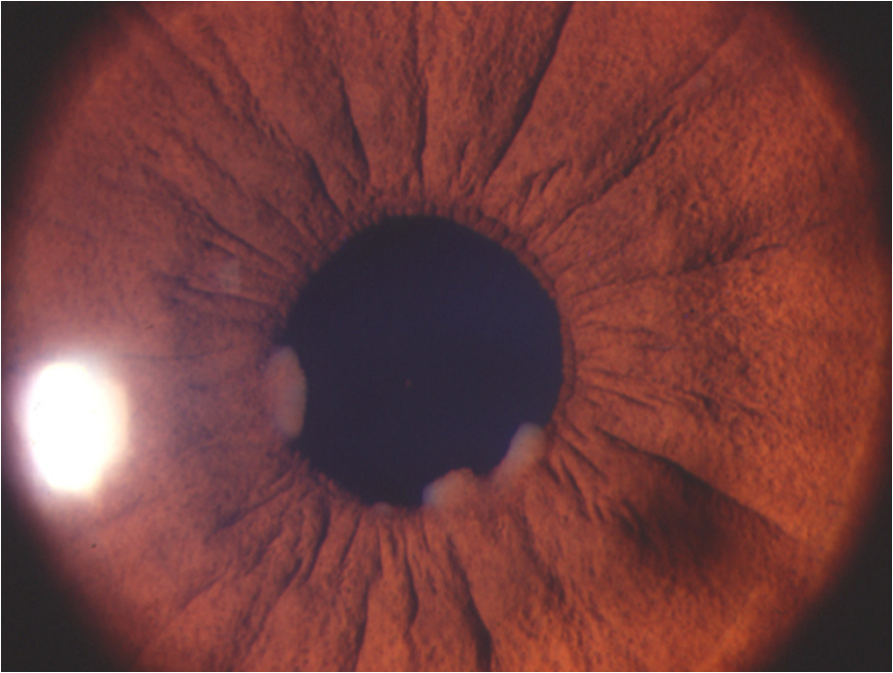
Slit lamp photograph showing the granulomatous anterior uveitis with large Koeppe nodules.

**Figure 4:**
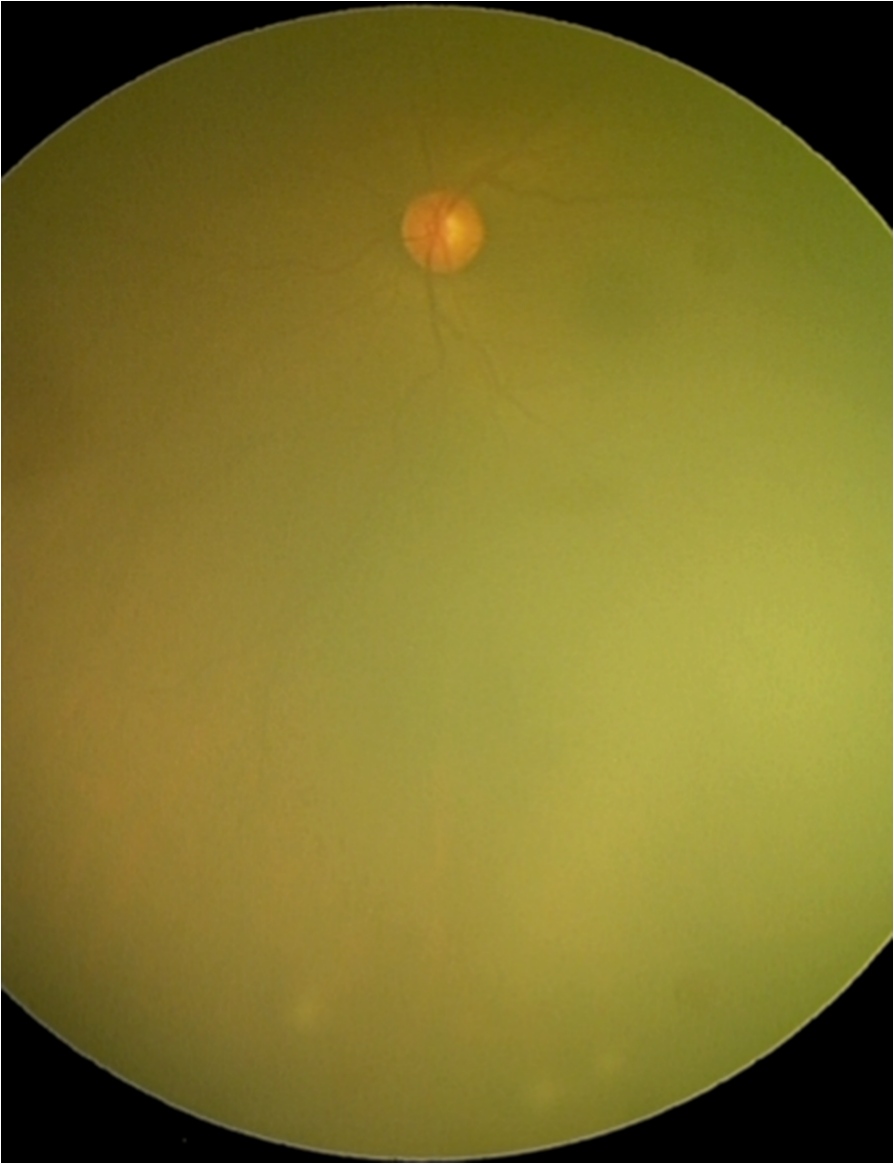
Fundus photograph showing vitritis with snow ball opacities in the peripheral retina.

**Figure 5:**
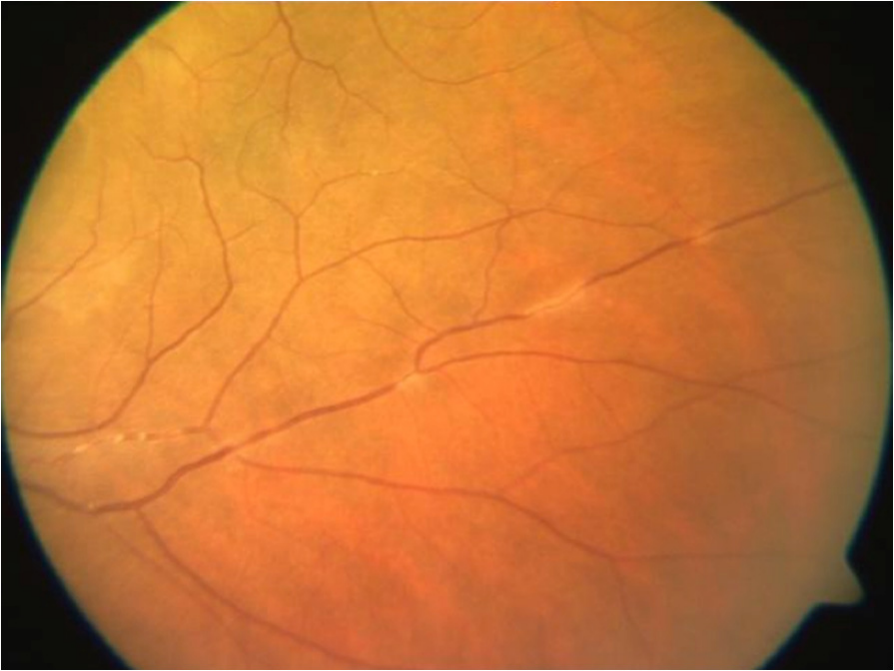
Fundus photograph showing the perivasculitis.

**Figure 6:**
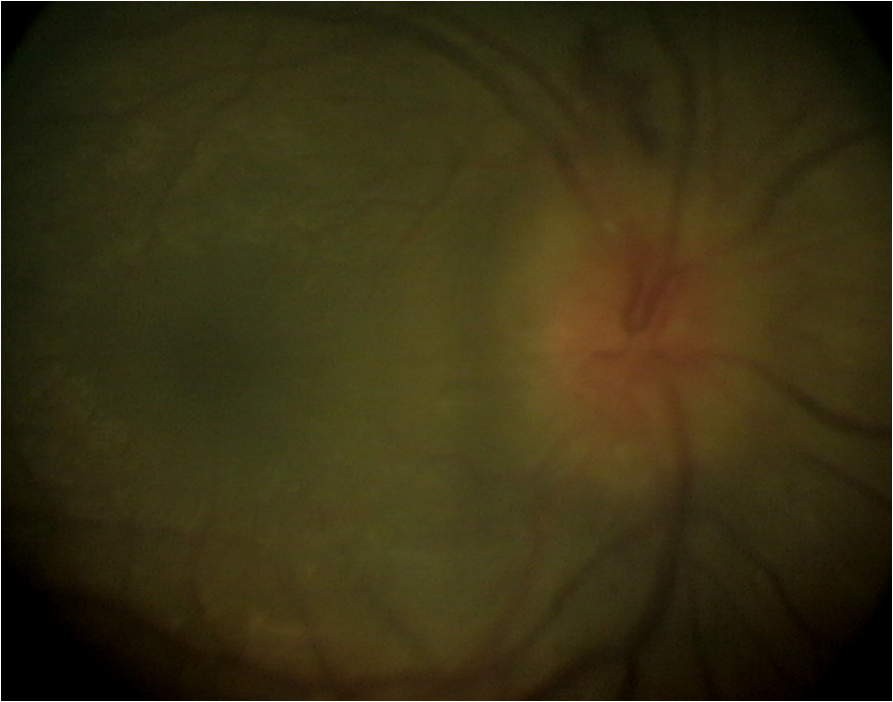
Fundus photograph showing the disc edema.

**Figure 7:**
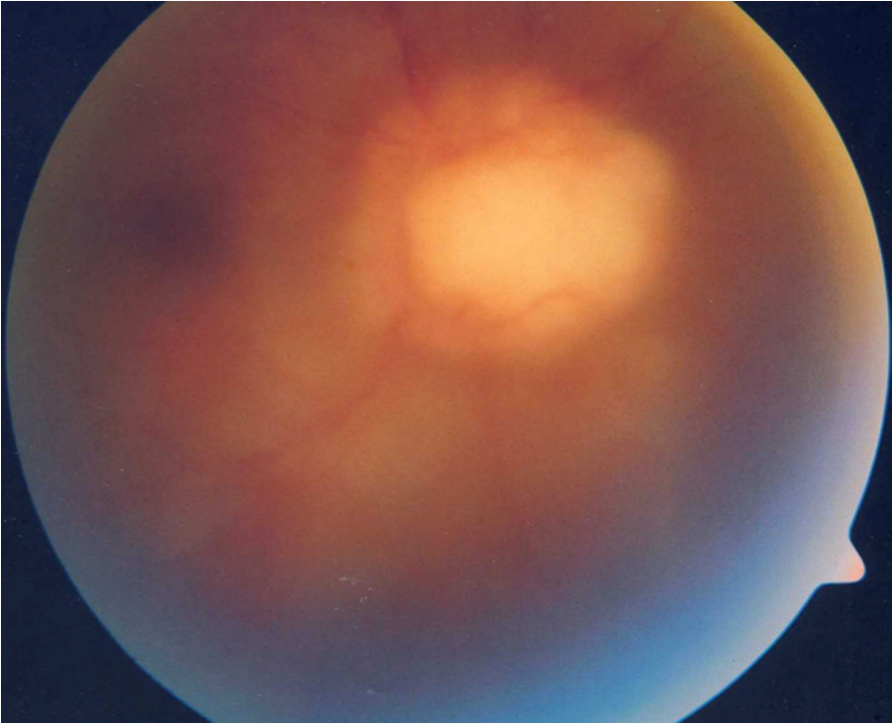
Fundus photograph showing the optic nerve granuloma.

**Figure 8:**
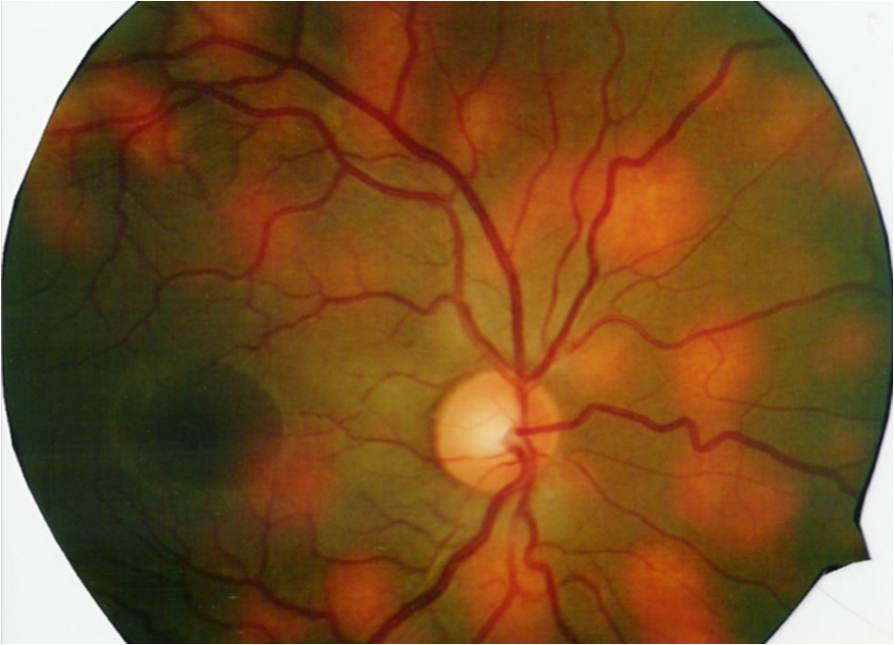
Fundus photograph showing the choroidal granulomas.

**Figure 9:**
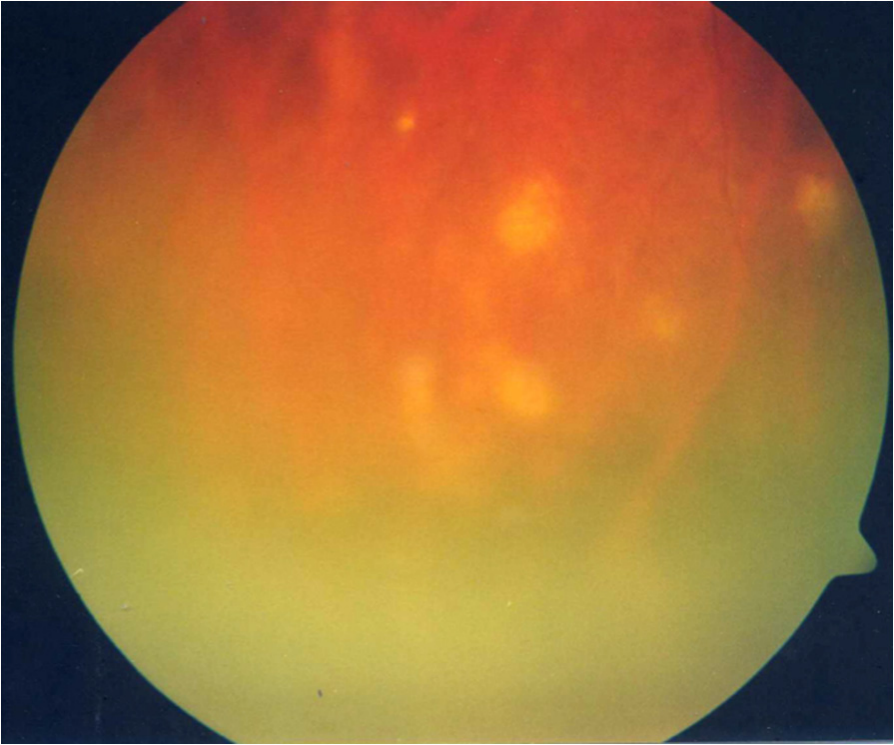
Fundus photograph showing the peripheral chorioretinal punched out scars in a patient with sarcoidosis.

**Figure 10:**
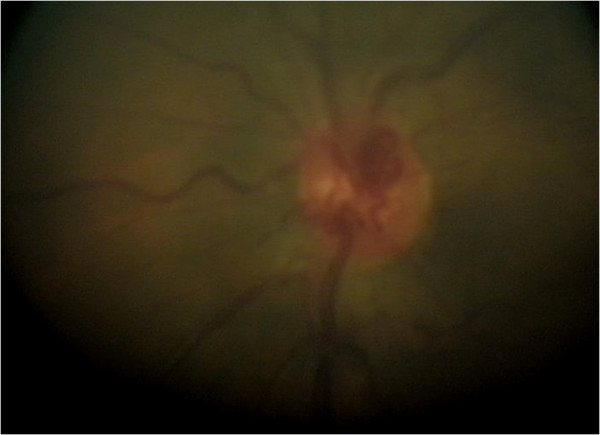
Fundus photograph showing the optic nerve head anastomosis in a patient with cutaneous sarcoidosis.

**Figure 11:**
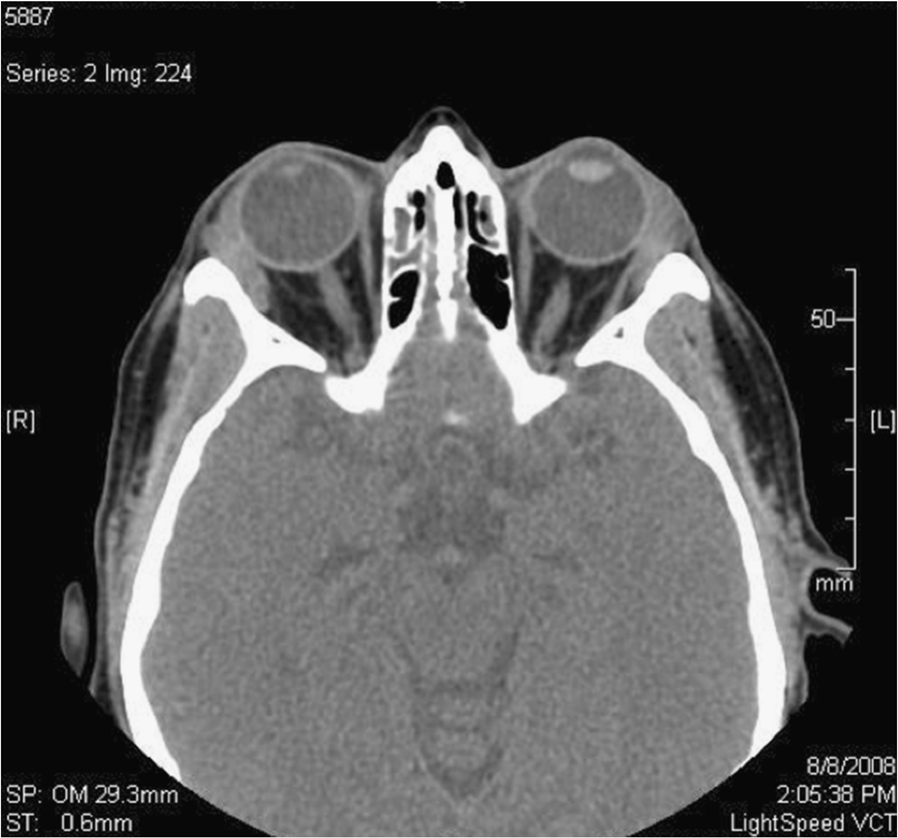
High resolution computed tomography of the orbits showing bilateral lacrimal gland enlargement.

**Figure 12:**
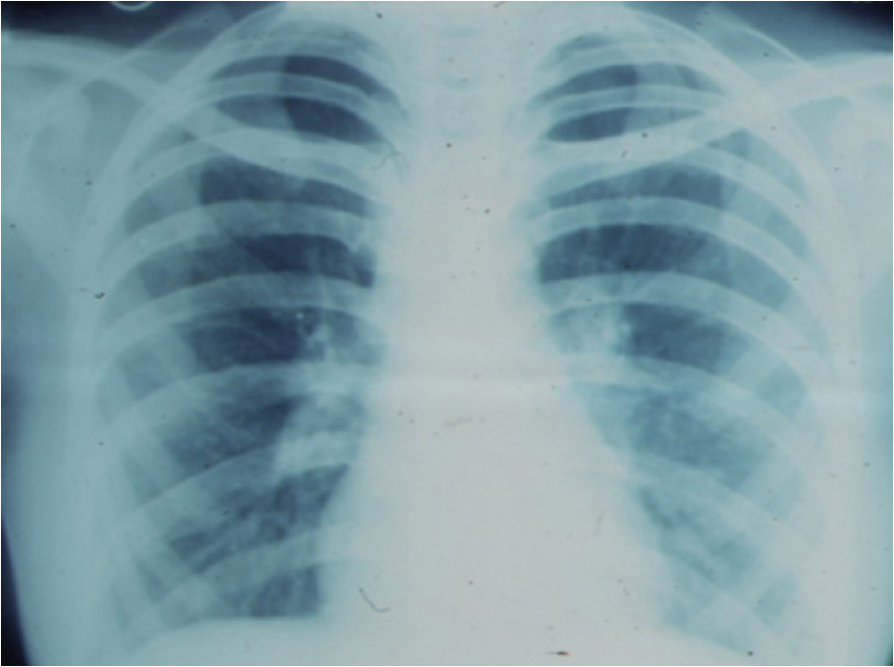
Chest X-ray showing bilateral hilar lymphadenopathy in a patient with sarcoidosis.

**Figure 13:**
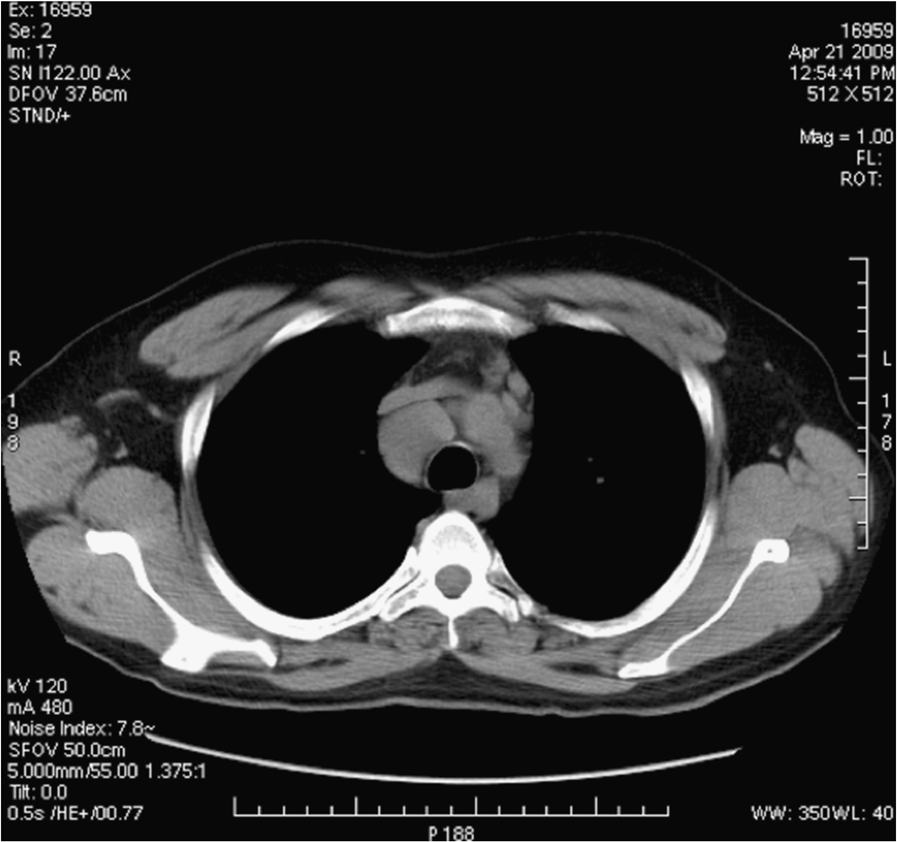
High resolution computed tomography of the thorax showing mediastinal lymphadenopathy in a patient with grade 2 sarcoid with respiratory symptoms.

**Figure 14:**
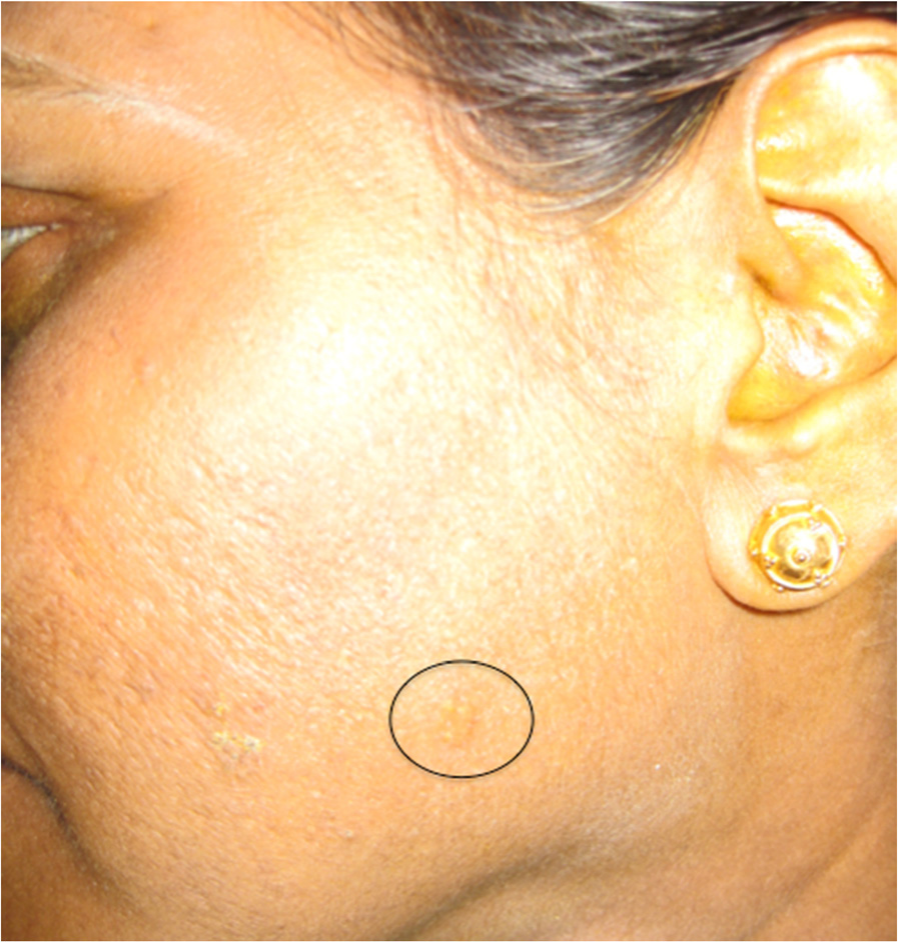
External photograph of sarcoid granuloma on the face.

**Figure 15:**
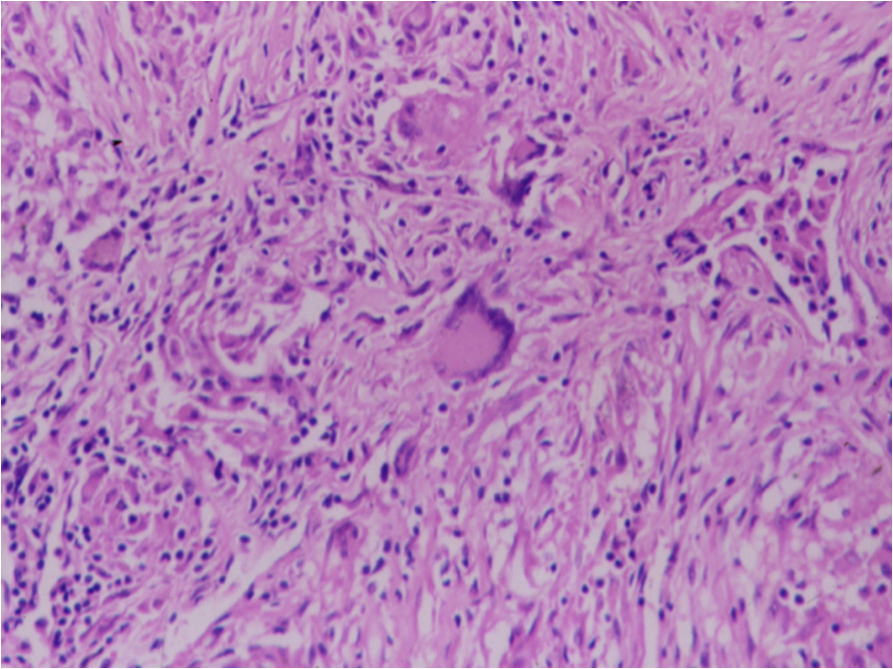
Photomicrograph of biopsy from lacrimal glands showing non-caseating granulomas with giant cells.

Anterior uveitis could be acute iridocyclitis or a chronic granulomatous uveitis with keratic precipitates, which may vary from cellular to large mutton fat type of keratic precipitates. Iris nodules such as the Koeppe and busacca nodules and iris granulomas may be seen. Granulomas may also be seen in the trabecular meshwork causing increase in intraocular pressure at the time of activity. Intermediate uveitis with vitritis, peripheral vasculitis, snowball opacities, and snow banking have been described in reports from India as well. Posterior segment involvement includes periphlebitis with characteristic ‘candle wax dripping appearances’ , multifocal chorioretinitis, peripheral punched out chorioretinal lesions, choroid and optic nerve granulomas, optic disc edema, papilledema, optociliary shunts, and arterial macro aneurysms. Rare manifestations of scleral nodule, vasoproliferative tumors, and retinitis have also been reported from India. Conjunctival granulomas, bilateral lacrimal gland enlargement, and orbital mass lesions have been reported from India [9,34]. Illustrations are provided as Figures 1, 2, 3, 4, 5, 6, 7 , 8 , 9, 10, 11, 12, 13, 14, and 15.

### Diagnostic modalities

A negative Mantoux (PPD) skin test is considered to suggest sarcoidosis and is usually seen in 90% of sarcoid patients [36]. Elevated serum angiotensin converting enzyme is elevated in only half the patients with sarcoidosis and may be also seen in tuberculosis and, hence, nonspecific. Bronchoalveolar lavage and gallium citrate scanning is also nonspecific and may be positive in tuberculosis as well.

High resolution CT is of great help in making the diagnosis of sarcoidosis by detecting interstitial involvement and lymphadenopathy. Contrast-enhanced CT may help distinguish sarcoid from tubercular lymph node enlargement, which would usually show a central hypodensity corresponding to the caseation and peripheral contrast enhancement. The frequent use of fiberoptic bronchoscopy with transbronchial lymph node and endobronchial lung biopsies with diagnostic yield of about 80% have helped in the recognition of sarcoidosis. Microbiology (smear and culture for MTB) and histopathology are routinely done on the aspirates/biopsy material to rule out TB.

### The sarcoid-TB dilemma

Both TB and sarcoid share remarkable similarities in their clinical and radiological presentations. Both diseases present with constitutional symptoms of fever, malaise, weight loss, and fatigue. Respiratory symptoms are common to both diseases. Similar ocular manifestations are seen in both diseases. Serpiginous-like choroiditis in the Indian subcontinent are more likely to be associated with tuberculosis [37]; however, rare case reports of sarcoidosis presenting with an APMPPE-like manifestation have also been reported [35]. Dry eye, bilateral lacrimal gland enlargements are more common in sarcoidosis, though latter have been reported in TB also [38]. In a study conducted by us in patients with biopsy-proven granulomatous uveitis, we reported that the likelihood ratio of the uveitis being tubercular is around 76.6%, if a combination of pigmented multifocal choroiditis patches along the retinal blood vessels, a Schirmer test ≥ 10 mm, and a positive Mantoux test are present [8].

Radiologic similarities may be seen in both diseases. Discrete, bilateral, symmetrical lymph node enlargement is usually seen in sarcoidosis. Fibrosis and military distribution although common in TB may be seen in both diseases. Both diseases show granulomatous inflammation on histopathology. Caseation is seen in tuberculosis. Fibrinoid necrosis though rare can occur in up to 30% of cases of sarcoidosis. There are no specific features enough to differentiate the two conditions with certainty, except a positive culture of MTb. A positive PCR for MTb may be seen in the biopsy samples of sarcoidosis.

Differentiating the two conditions pose a challenge, particularly in countries with high prevalence of TB. A combination of clinical, radiologic, and laboratory tests are used to make a diagnosis of sarcoidosis. Tuberculin skin test plays an important role in the diagnosis [36]. Tuberculin anergy is a clinically important phenomenon in sarcoidosis. As a negative tuberculin test excludes TB except in immunosuppressed or extremely sick individuals, we do attach a great deal of importance to this test. On the other hand, patients with positive interferon-γ release assays (IGRA) are sometimes misdiagnosed as TB. A recent study from India shows positive interferon-γ release assay in some patients with sarcoidosis. Thus, in countries with high TB burden, one must not make a diagnosis of TB on the basis of positive IGRA alone [6].

### Treatment

Corticosteroids are the mainstay of treatment in sarcoidosis. In chronic cases or in those patients were steroids are contraindicated, immunosuppressants like methotrexate, azathioprine, mycophenolate mofetil, cyclosporine, and cyclophosphamide have been used [39,40]. Methotrexate has been used most frequently, as it is cost effective. Aggressive cases may even require intravenous methyl prednisolone or pulse intravenous cyclophosphamide. Use of biologics like rituximab [41] in steroid-resistant cases of sarcoid has been reported. Many times, patients are treated with antitubercular therapy prior to the diagnosis of sarcoid.

## Conclusions

Diagnosing sarcoid in countries with high TB burden does pose a significant challenge. However, new cases of sarcoid are increasingly diagnosed in TB endemic areas in recent years due to increased awareness and better availability of diagnostic modalities. Ocular evaluations do contribute in making a diagnosis of systemic sarcoid. Negative Mantoux test is still being considered an important diagnostic test to make a diagnosis. Sarcoidosis does exist in populations with high TB burden, and the association between sarcoid and TB continues to be an enigma. Prospective studies from the Indian continent on the epidemiological data, genotypic and phenotypic variations, and the role of mycobacteria in the development of sarcoidosis would contribute a great deal in understanding this disease.

### Consent

Written informed consent was obtained from the patient for the publication of this report and any accompanying images. The consent to publish has been provided by the patient with the face photograph and has been approved by our Institutional Review Board (Vittala International Institute of Ophthalmology).

## Competing interest

The author declares that she has no competing interests.

## References

[B1] Newman LS Rose CS Maier LA Sarcoidosis N Engl J Med 1997 3 1223 1234 10.1056/NEJM199704243361705 9110911

[B2] Hosoda Y Kosuda T Yamamoto M Hongo O Mochizumi H Mikami R Homma H Fujita S Ohira I Izumi T Kobara Y Yammato H Oshima S Teramatsu T Maekawa N Tsuji S Soon CP Sodhy TS Bovornkitti S Chakravarty SC Yang SP Gomaa T A cooperative study of sarcoidosis in Asia and Africa: descriptive epidemiology Ann N Y Acad Sci 1976 3 347 354 10.1111/j.1749-6632.1976.tb47045.x 1067019

[B3] Jindal SK Gupta D Aggarwal AN Sarcoidosis in developing countries Curr Opin Pulm Med 2000 3 5 448 454 10.1097/00063198-200009000-00011 10958238

[B4] Gupta D Agarwal R Aggarwal AN Jindal SK Sarcoidosis and tuberculosis: the same disease with different manifestations or similar manifestations of different disorders Curr Opin Pulm Med 2012 3 5 506 516 10.1097/MCP.0b013e3283560809 22759770

[B5] Gupta D Agarwal R Aggarwal N Jindal SK Molecular evidence for the role of mycobacteria in sarcoidosis: a meta analysis Eur Respir J 2007 3 508 516 10.1183/09031936.00002607 17537780

[B6] Gupta D Kumar S Aggarwal AN Verma I Agarwal R Interferon gamma release assay (QuantiFERON-TB Gold In Tube) in patients of sarcoidosis from a population with high prevalence of tuberculosis infection Sarcoidosis Vasc Diffuse Lung Dis 2011 3 2 95 101 22117500

[B7] Kumar R Goel N Gaur SN Sarcoidosis in north Indian population: a retrospective study Indian J Chest Dis Allied Sci 2012 3 2 99 104 22973778

[B8] Babu K Kini R Mehta R Philips M Subbakrishna DK Murthy KR Predictors for tubercular uveitis: a comparison between biopsy-proven cases of tubercular and sarcoid uveitis Retina 2012 3 5 1017 1020 10.1097/IAE.0b013e31822d3a20 22146129

[B9] Babu K Kini R Mehta R Abraham MP Subbakrishna DK Murthy KR Clinical profile of ocular sarcoidosis in a South Indian patient population Ocul Immunol Inflamm 2010 3 5 362 369 10.3109/09273948.2010.495813 20735280

[B10] Khanna A Sidhu U Bajwa G Malhotra V Pattern of ocular manifestations in patients with sarcoidosis in developing countries Acta Ophthalmol Scand 2007 3 6 609 612 10.1111/j.1600-0420.2006.00791.x 17651463

[B11] Mootha VK Agarwal R Aggarwal AN Gupta D Ahmed J Verma I Bal A The sarcoid-tuberculosis link: evidence from a high TB prevalence country J Infect 2010 3 6 501 503 10.1016/j.jinf.2010.03.010 20346973

[B12] Agarwal R Aggarwal AN Gupta D Efficacy and safety of conventional TBNA in sarcoidosis: a systematic review and meta-analysis Respir Care 2012 in press 10.4187/respcare.02101 23050747

[B13] Sharma SK Mohan A Sarcoidosis in India: not so rare J Indian Acad Clin Med 2004 3 1 12 21

[B14] Anantham D Ong SJ Chuah KL Fook-Chong S Hsu A Eng P Sarcoidosis in Singapore: epidemiology, clinical presentation and ethnic differences Respirology 2007 3 3 355 360 10.1111/j.1440-1843.2007.01074.x 17539838

[B15] Liam CK Menon A Sarcoidosis: a review of cases seen at the university hospital, Kuala Lumpur Singapore Med J 1993 3 2 153 156 8266159

[B16] Pathanapitoon K Goossens JH van Tilborg TC Kunavisarut P Choovuthayakorn J Rothova A Ocular sarcoidosis in Thailand Eye (Lond) 2010 3 11 1669 1674 10.1038/eye.2010.107 20689569

[B17] Sheu SJ Chang FP Wu TT Chuang CT Ocular sarcoidosis in southern Taiwan Ocul Immunol Inflamm 2010 3 3 152 157 10.3109/09273941003637502 20482387

[B18] Babu K Kini R Mehta R Scleral nodule and bilateral disc edema as a presenting manifestation of systemic sarcoidosis Ocul Immunol Inflamm 2010 3 3 158 161 10.3109/09273941003753416 20482388

[B19] Biswas J Krishnakumar S Raghavendran R Mahesh L Lid swelling and diplopia as presenting features of orbital sarcoid Indian J Ophthalmol 2000 3 3 231 233 11217257

[B20] Shabbir Shafiq J Biswas J Optic nerve head sarcoid granuloma treated with intravenous methyl prednisolone Oman J Ophthalmol 2008 3 1 28 31 10.4103/0974-620X.43318

[B21] Verma A Biswas J Choroidal granuloma as an initial manifestation of systemic sarcoidosis Int Ophthalmol 2010 3 5 603 606 10.1007/s10792-009-9328-5 19998054

[B22] Ganesh SK Roopleen BJ Veena N Role of high-resolution computerized tomography (HRCT) of the chest in granulomatous uveitis: a tertiary uveitis clinic experience from India Ocul Immunol Inflamm 2011 3 1 51 57 10.3109/09273948.2010.525680 21250925

[B23] Brownell I Ramirez-Valle F Sanchez M Prystowsky S Evidence for mycobacteria in sarcoidosis Am J Respir Cell Mol Biol 2011 3 899 905 10.1165/rcmb.2010-0433TR 21659662 PMC3361363

[B24] Scadding JG Mycobacterium tuberculosis in the aetiology of sarcoidosis Br Med J 1960 3 1617 1623 10.1136/bmj.2.5213.1617 13747016 PMC2098450

[B25] Drake WP Newman LS Mycobacterial antigens may be important in sarcoidosis pathogenesis Curr Opin Pulm Med 2006 3 359 363 10.1097/01.mcp.0000239554.01068.94 16926652

[B26] Parsons V Awareness of family and contact history of tuberculosis in generalized sarcoidosis Br Med J 1960 3 1756 1759 10.1136/bmj.2.5215.1756 13733025 PMC2098498

[B27] Hosoda Y Sasagawa S Yamaguchi T Sarcoidosis and tuberculosis: epidemiological similarities and dissimilarities. A review of a series of studies in a Japanese work population (1941–1996) and the general population (1959–1984) Sarcoidosis Vasc Diffuse Lung Dis 2004 3 2 85 93 15281429

[B28] Hosoda Y Yamaguchi M Hiraga Y Global epidemiology of sarcoidosis. What story do prevalence and incidence tell us? Clin Chest Med 1997 3 4 681 694 10.1016/S0272-5231(05)70412-3 9413652

[B29] Drake WP Newman LS Mycobacterial antigens may be important in sarcoidosis pathogenesis Curr Opin Pulm Med 2006 3 5 359 363 10.1097/01.mcp.0000239554.01068.94 16926652

[B30] Agarwal R Gupta D Srinivas R Verma I Aggarwal AN Laal S Analysis of humoral responses to proteins encoded by region of difference 1 of mycobacterium tuberculosis in sarcoidosis in a high tuberculosis prevalence country Indian J Med Res 2012 3 6 920 923 22825614 PMC3410222

[B31] Chan AS Sharma OP Rao NA Review for disease of the year: immunopathogenesis of ocular sarcoidosis Ocul Immunol Inflamm 2010 3 3 143 151 10.3109/09273948.2010.481772 20482386

[B32] Song Z Marzilli L Greenlee BM Chen ES Silver RF Askin FB Teirstein AS Zhang Y Cotter RJ Moller DR Mycobacterial catalase-peroxidase is a tissue antigen and target of the adaptive immune response in systemic sarcoidosis J Exp Med 2005 3 5 755 767 Erratum in: J Exp Med. 2005; 202(5):721 10.1084/jem.20040429 15753209 PMC2212832

[B33] Chen ES Wahlström J Song Z Willett MH Wikén M Yung RC West EE McDyer JF Zhang Y Eklund A Grunewald J Moller DR T cell responses to mycobacterial catalase-peroxidase profile a pathogenic antigen in systemic sarcoidosis J Immunol 2008 3 12 8784 8796 19050300 10.4049/jimmunol.181.12.8784 PMC2700300

[B34] Kim UR Khazaei H Stewart WB Shah AD Spectrum of orbital disease in South India: an Aravind study of 6328 consecutive patients Ophthal Plast Reconst Surg 2010 3 5 315 322 10.1097/IOP.0b013e3181c32f2f 20592641

[B35] Rothova A Ocular involvement in sarcoidosis Br J Ophthalmol 2000 3 1 110 116 10.1136/bjo.84.1.110 10611110 PMC1723211

[B36] Smith-Rohrberg D Sharma SK Tuberculin skin test among pulmonary sarcoidosis patients with and without tuberculosis: its utility for the screening of the two conditions in tuberculosis-endemic regions Sarcoidosis Vasc Diffuse Lung Dis 2006 3 2 130 134 17937109

[B37] Bansal R Gupta A Gupta V Dogra MR Sharma A Bambery P Tubercular serpiginous-like choroiditis presenting as multifocal serpiginoid choroiditis Ophthalmology 2012 3 11 2334 2342 10.1016/j.ophtha.2012.05.034 22892153

[B38] Bansal RK Malhotra C Bhatia R Chhabra S Sood S Tubercular dacryoadenitis–a case report and review of literature Indian J Pathol Microbiol 2006 3 3 385 387 17001891

[B39] Baughman RP Lower EE Ingledue R Kaufman AH Management of ocular sarcoidosis Sarcoidosis Vasc Diffuse Lung Dis 2012 3 1 26 33 23311120

[B40] Bhat P Cervantes-Castañeda RA Doctor PP Anzaar F Foster CS Mycophenolate mofetil therapy for sarcoidosis-associated uveitis Ocul Immunol Inflamm 2009 3 3 185 190 10.1080/09273940902862992 19585361

[B41] Lower EE Baughman RP Kaufman AH Rituximab for refractory granulomatous eye disease Clin Ophthalmol 2012 3 1613 1618 23055686 10.2147/OPTH.S35521 PMC3468281

